# Prevalence, Patterns, and Predictors of Oral Morbidity in Patients With Diabetes: Evidence From the Longitudinal Ageing Study in India

**DOI:** 10.7759/cureus.72164

**Published:** 2024-10-22

**Authors:** Shubhanjali Roy, Mansi Malik, Saurav Basu

**Affiliations:** 1 Dentistry, Indian Institute of Public Health - Delhi, Public Health Foundation of India, New Delhi, IND; 2 Clinical Research, Indian Institute of Public Health - Delhi, Public Health Foundation of India, New Delhi, IND; 3 Epidemiology and Public Health, Indian Institute of Public Health - Delhi, Public Health Foundation of India, New Delhi, IND

**Keywords:** dental screening, diabetes mellitus, epidemiology and public health, oral health awareness, oral manifestation of systemic diseases

## Abstract

Background

Diabetes mellitus (DM) and periodontal disease share a complex bidirectional relationship, resulting in worsening of oral health with persistent impairment of glycemic control.

Objective

The objective of this study was to ascertain the burden, patterns, and predictors of oral morbidities in older patients with DM, including their health-seeking behavior in India.

Materials and methods

We used the nationally representative Longitudinal Ageing Study in India (LASI) wave-1 dataset (2017) to analyze data from 8,564 patients with DM aged ≥45 years.

Results

The median duration of DM in the participants was eight years. The weighted prevalence of at least one or more self-reported oral health morbidities was 59.85% (95% CI: 56.57% to 63.13%) including tooth pain (33.47%), loose teeth (29.98%), swelling in gums (10.08%), and bleeding gums (9.1%). Furthermore, patients with DM had a substantial burden of total (8.84%) and partial edentulism (66.35%). On adjusted analysis, female gender, higher educational status, higher wealth quintile, tobacco use, alcohol use, and greater duration of DM had significantly higher odds of having oral health morbidities excluding caries. Only 79 (0.12%) patients reported visiting dentists in the previous 12 months.

Conclusions

Integration of oral healthcare services with existing diabetes care in outpatient settings should be strengthened to improve oral health related quality of life.

## Introduction

Diabetes mellitus (DM) is a chronic metabolic illness marked by high blood sugar levels due to underlying dysfunction of insulin secretion, action, or both, and is a major public health concern, with rapidly increasing prevalence worldwide [1,2]. DM is associated with several complications, including cardiovascular disease, renal failure, neuropathy, and amputations, that contribute to increased morbidity and mortality rates apart from the economic costs associated with healthcare and reduced economic productivity. The burden of DM is highest in low- and middle-income countries with resource-limited health systems, further aggravating health inequities.

The International Diabetes Federation estimates that 463 million persons worldwide had DM in 2019, and if current trends continue, this figure is expected to rise to 700 million by 2045 [3]. India has the second highest global burden of DM globally, with nearly 11% of its population estimated as having DM [4].

DM also has deleterious oral health consequences, including an increased risk of periodontal disease, dental caries, oral infections, slow wound healing, burning mouth syndrome, taste impairment, and salivary gland dysfunction, highlighting the need for people with DM to prioritize oral health. Approximately 22% of individuals with DM experience periodontal disease, which can be more severe in those with poor blood sugar control [5]. The risk of dental caries is notably elevated, with studies indicating that individuals with DM may experience higher rates of tooth decay due to factors such as dry mouth and high glucose levels in saliva. Slow wound healing is also a common issue, stemming from impaired blood flow and immune response. Burning mouth syndrome affects around 20% of this population, while taste impairment is reported in approximately 15% of individuals, often linked to neuropathy or medication side effects [6]. Furthermore, around 30% may experience salivary gland dysfunction, leading to dry mouth (xerostomia), which can exacerbate other oral health complications [5]. There is growing recognition of the reciprocal interaction between DM and oral morbidity having a bidirectional relationship that worsens oral health and glycemic control in a vicious cycle [6].

The prevalence of periodontal disease is notably high in patients with DM, with studies showing that around 59.7% of African Americans with type 2 DM mellitus (T2DM) experience this condition [7]. It is previously estimated from global data through a systematic review that 90% of patients with DM have oral manifestations [8]. Oral health problems affect all groups and negatively impact general health and wellness, although there is mixed evidence on whether oral health related quality of life is causally linked to DM or only a sequelae of oral health complications in patients with DM [9-12].

DM burden is suspected to accentuate the risk of oral morbidities through multiorgan changes and associated immunological alterations, although the relationship is not completely understood [12]. Epidemiological studies also indicate that Latent Autoimmune Diabetes in Adults (LADA) accounts for approximately 2% to 12% of all adult-onset DM cases, with variations depending on geographic and ethnic factors. A systematic review found a global prevalence of LADA at 8.9% in adult-onset diabetic individuals, with rates ranging from 2.3% in some regions to as high as 18.9% in others, such as Bahrain and the United Arab Emirates [13].

Oral healthcare is neglected not just at the individual level but also within health programs, policies, and healthcare insurance, which can negatively impact the attainment of the targets of Universal Health Coverage [14]. Furthermore, in India, most of the population is dependent on private dental services, which are usually not covered under insurance, resulting in high out-of-pocket expenses that undermine effective dental health-seeking behavior, with dental visits primarily directed for cosmetic procedures or urgent care and rarely for preventive services [15,16].

The lack of integration of oral health with noncommunicable disease (NCD) programs in India precludes effective management of oral morbidities in the high-risk older adults having DM group [17,18]. However, till date, there is sparse information on oral health status in patients with DM in India, which is mostly obtained from tertiary care facilities, and thus findings may not be generalizable to communities with limited accessibility to healthcare facilities [19].

We therefore conducted this study with the objective of ascertaining the burden and patterns of dental morbidities, and their predictors in older patients with DM, and health-seeking behavior from nationally representative community data from India. The information from this study would be useful for strengthening evidence-based linkages between the National Programme for Non-Communicable Diseases (NP-NCD) and the National Oral Health Programme.

## Materials and methods

Study design and data source

This secondary data analysis examined the Longitudinal Ageing Study in India (LASI) wave-1 dataset (2017) that collected health data of the older adult population from 35 Indian states and union territories, offering national representation. LASI was established under the guidance of the Ministry of Health and Family Welfare, Government of India, and implemented in collaboration with the International Institute for Population Sciences (IIPS), Harvard T.H. Chan School of Public Health, and others [18]. Notably, LASI included data of all residents aged 45 years or older, as well as their spouses, regardless of age. LASI employed a multi-stage stratified regions probability cluster sampling design, the details of which have been reported [20].

Each eligible individual participated in three survey schedules (household, individual, and community schedules) through face-to-face community-based interviews. To ensure consistency and uniformity across all states, field investigators and supervisors received comprehensive training at both national and state levels. They were equipped with a survey interview manual, one of five provided manuals, which contained detailed instructions for conducting interviews, question-by-question protocols, outcome measures, and a thorough discussion of the questionnaires. The survey interview manual not only clarified the purpose of each question but also provided guidance on how to ask the questions, probe for answers, and accurately record response codes using CAPI (a mini laptop used for data collection) [20].

Study sample

The sample for this current study included the subset of patients ever diagnosed with DM, i.e., individuals who responded “yes” to the following question were retained: “Has any health professional ever diagnosed you with diabetes or high blood sugar? - Yes/ No.”

Outcome variable

Participants with DM responding “yes” to any one of the oral morbidities were considered as having oral morbidity and were coded as “1,” whereas those participants with DM responding “no” to all the oral morbidities were coded as “0” for not having any oral morbidity. The exact question used in the survey was as follows:

In the last 12 months, have you ever been diagnosed with or suffered from any of the following oral problem(s)?

1. Painful teeth, 2. Ulcers lasting more than two weeks, 3. Bleeding gums. 4. Swelling gums. 5. Loose teeth. 6. Dental cavity/dental caries. 7. Soreness or cracks in the corner of the mouth. 8. Others.

or

Have you lost some or all of your natural teeth?

1. Yes, lost all natural teeth. 2. Yes, lost some natural teeth. 3. No, have not lost any teeth.

Independent variables

Sociodemographic and behavioral covariates were considered in this analysis. Age was categorized as 45-59, 60-74, and ≥75 years, while sex was categorized as “male” and “female.” Education was categorized as “less than primary,” “less than secondary,” “primary,” “middle/ secondary,” “high secondary” and “graduate and above.” Marital status was categorized as “unmarried,” “married/ live-in,” while the rest were clubbed into the third category: “divorced/separated/widow/deserted.” Wealth index/monthly per capita expenditure (MPCE) had five categories, ranging from “poorest” to “richest.” Tobacco Usage was categorized as “do not consume tobacco,” “smoke tobacco,” “smokeless tobacco,” and “both smoke and smokeless tobacco.” Alcohol consumption was categorized as “does not consume alcohol,” “occasionally,” and “weekly.” Presence of health insurance was categorized as “yes” and “no.” Intake of insulin shots by the individuals was categorized as “yes” and “no.” Special diet for DM included dichotomized “yes” and “no” responses.

Health-seeking behavior of the respondents was assessed based on the following variables

Type of health facility accessed: It was categorized as “public facility,” “private facility,” “AYUSH (Ayurveda Yoga & Naturopathy, Unani, Siddha and Homeopathy) facility belonging to the Indian Systems of Medicines,” and “others,” which included pharmacists and homecare.

Type of provider accessed: It was categorized as “doctor,” “dentist,” “AYUSH practitioner,” “nurse/pharmacists” and “others.”

Statistical analysis

Data were analyzed using STATA 15.1 (StataCorp LLC, College Station, TX). Weights were applied throughout the analysis of the LASI dataset to account for the impact of the multi-stage sampling strategy. For categorical variables and to represent the prevalence of outcome variables, weighted frequencies and percentages were used. Bivariate analysis was conducted using the chi-square test to ascertain association between oral morbidities and the independent variables. The crude odds ratios (OR), adjusted odds ratios (aOR), along with their respective 95% confidence intervals (CI), and p-values were reported.

The adjusted model included variables that were statistically significant, along with variables reported to be associated with oral morbidities in the existing literature. Additionally, the linearity of logit, representing the relationship between the log odds (logit) of the outcome variable and the predictor variable, was assessed. A p-value of <0.05 was considered statistically significant. Furthermore, the weighted prevalence of different co-occurring oral morbidities within participants with DM was computed.

Ethical considerations

The study uses secondary data, which are available on reasonable request through https://www.iipsindia.ac.in/content/lasi-wave-i. Before participating in the study, all participants were provided with information about the research, and their consent was obtained through signed informed consent forms. The original LASI study received approval from ICMR (Indian Council of Medical Research), Delhi, and IIPS (International Institute of Population Sciences), Mumbai, along with other affiliated bodies.

## Results

The study sample included information from 8,564 individuals with mean (SD) age of 61.73 (9.88) years. The sociodemographic characteristics of the participants are reported in Table 1.

**Table 1:** Sociodemographic description of patients with diabetes mellitus. AYUSH, Ayurveda Yoga & Naturopathy, Unani, Siddha and Homeopathy

Variables	Total (N)	%
Age (years)		
45-59	3,630	42.08%
60-74	3,994	47.21%
≥75	940	10.70%
Sex		
Male	4,122	46.40%
Female	4,442	53.59%
Educational qualification		
No formal education	1,011	17.32%
Primary	1,333	21.66%
Middle/secondary	2,169	35.60%
High school	575	8.98%
Graduate and above	843	16.43%
Marital status		
Unmarried	70	0.54%
Married/ live in	6,511	74.51%
Separated/divorced/widow	1,983	24.95%
Wealth index		
Poorest	1,158	14.28%
Poorer	1,401	15.70%
Middle	1,665	18.25%
Richer	1,982	23.12%
Richest	2,358	28.62%
Frequency of alcohol		
Does not consume alcohol	7,317	88.28%
Occasionally	990	9.70%
Weekly	250	2.01%
Type of tobacco		
Do not consume tobacco	6,312	74.45%
Smoke tobacco	921	9.63%
Smokeless tobacco	1,150	13.57%
Both smoke and smokeless	190	2.34%
Health insurance		
No	6,543	77.70%
Yes	1,947	22.93%
Caste		
Scheduled caste	1,113	14.10%
Scheduled tribe	873	3.65%
Other backward class	3,479	53.19%
Others	2,713	29.05%
Residence		
Rural	3,747	43.75%
Urban	4,817	56.25%
Duration DM (years), median (IQR)	8 (6, 13)	
DM medication		
No	1,562	18.56%
Yes	7,000	81.44%
Taking insulin shots		
No	7,451	83.02%
Yes	1,108	16.98%
Special diet for DM		
No	2,089	24.33%
Yes	6,469	75.67%
Health-seeking behavior		
OPD visits in the past 12 months		
No		71.74
Yes		28.26
Type of health facility accessed		
Public		55.35
Private		22.81
AYUSH		2.03
others		19.82
Type of provider accessed		
Medical doctor		86.38
Dentist		0.12
AYUSH practitioner		2.52
Nurse/pharmacist/others		10.98

A majority of the participants (57.91%) were older adults. There were 46.40% male and 53.59% female participants, respectively. A majority (61.1%) of the participants were educated beyond elementary school. As per the wealth index, the richest participants comprised 28.62% and the poorest comprised 14.28% of the participants. The vast majority of participants did not use tobacco or alcohol (74.45%) or engage in other health-related behaviors (88.28%). In addition, 9.63% of individuals who used tobacco did so by smoking, 13.57% by using smokeless tobacco, and 2.34% by using both. A majority of the participants (56.25%) lived in urban areas, while the remainder (43.75%) lived in rural areas. The median (IQR) duration of DM was 8 (6, 13) years, with 81.44% patients on oral hypoglycemic agents and 16.98% on insulin. Outpatient care services were availed by 28.26% of the participants in the previous 12 months. The majority of participants sought healthcare from public facilities (55.35%), followed by private facilities (22.81%). Only 0.12% (n=79) of participants reported specifically visiting dentists, of which 89.87% (n=71) had oral morbidities.

Patterns of oral comorbidities in patients with DM are reported in Table 2.

**Table 2:** Weighted prevalence of various oral comorbidities in patients with diabetes mellitus.

Oral morbidities	N (%)	95% CI
Painful teeth	2,443 (33.47)	(29.12, 38.14)
Ulcers lasting more than two weeks	179 (2.20)	(1.79, 2.70)
Bleeding gums	571 (8.91)	(6.28, 12.52)
Swelling gums	855 (10.08)	(8.97, 11.32)
Loose teeth	2,350 (29.98)	(26.27, 33.69)
Dental cavity/caries	1,970 (20.43)	(17.41, 23.82)
Soreness or cracks in the corner of the mouth	343 (7.44)	(4.22, 12.80)
Complete edentulism	646 (8.84)	(6.24, 12.37)
Partial edentulism	5,691 (66.35)	(62.85, 69.67)
Clusters		
Painful teeth + bleeding gums	430	7.03 (4.55, 10.72)
Painful teeth + swelling gums	629	7.50 (6.58, 8.53)
Painful teeth + loose teeth	097	15.34 (11.76, 19.76)
Painful teeth + dental caries	981	11.08 (8.43, 14.43)
Painful teeth + swelling gums + bleeding gums	268	3.62 (3.01, 4.36)
Bleeding gums + swelling gums	347	4.59 (3.90, 5.40)
Bleeding gums + loose teeth	316	5.28 (2.96, 9.25)
Swelling gums + loose teeth	427	4.91 (4.24, 5.69)
Swelling gums + dental caries	297	2.89 (2.42, 3.44)
Loose teeth + dental caries	883	10.72 (8.01, 14.21)

The combination of tooth pain and loose teeth was observed in 15.34% (95% CI: 11.76, 19.76) participants. The weighted prevalence of at least one or more oral health morbidities in the participants was 59.85% (95% CI: 56.57% to 63.13%). The major morbidities included tooth pain, reported by 33.47% (95% CI: 29.12, 38.14) participants, loose teeth (29.98%; 95% CI: 26.27, 33.69), dental caries or cavities (20.43%; 95% CI: 17.41, 23.82), swelling in gums (10.08%; 95% CI: 8.97, 11.32), bleeding gums (9.1%; 95% CI: 6.28, 12.52), soreness or cracks in the mouth corners, (7.44%; 95% CI: 4.22, 12.80), and ulcers lasting longer than two weeks (2.20%; (95% CI: 1.79, 2.70). Furthermore, patients with DM had a substantial burden of total edentulism (8.84%; 95% CI: 6.24, 12.37) and partial edentulism (66.35%; 95% CI: 62.85, 69.67).

A binary logistic regression analysis was conducted to ascertain the sociodemographic factors that were independently associated with oral morbidities (excluding caries) in the participants. The variables having significantly higher odds of poor oral health in the participants include female compared to male gender (aOR 1.48; 95% CI: 1.14, 1.92; p = 0.003), highest wealth compared to other wealth quintiles (aOR 1.51; 95% CI: 1.01, 2.26; p = 0.04), occasional alcohol drinkers compared to non-drinkers (aOR 1.34; 95% CI: 1.01, 1.80; p = 0.04), tobacco smokers compared to non-smokers (aOR 1.41; 95% CI: 1.08, 2.06; p = 0.02), longer than median duration of DM (8 years) compared to shorter duration since diagnosis of DM (aOR 1.41; 95% CI: 1.07, 1.85; p = 0.015), and insulin users compared to OHA users (aOR 2.17; 95% CI: 1.28, 3.67; p = 0.0039). However, lower educational status was protective against poor oral health (aOR 0.58; 95% CI: 0.38, 0.88; p = 0.01). However, factors such as age, availability of any health insurance, consumption of a specific diet, place of residence, and social caste were not statistically significant (Table 3).

**Table 3:** Logistic regression analysis of the factors associated with poor oral health in patients with diabetes mellitus.

Variables	Crude OR (95% CI)	p-Value	Adjusted OR (95% CI)	p-Value
Age (in years)				
45-59	1	0.2674	1	
60-74	1.27 (0.94, 1.71)		1.17 (0.88, 1.56)	0.28
≥75	1.26 (0.86, 1.84)		1.08 (0.67, 1.75)	0.74
Sex				
Male	1	0.003	1	
Female	1.48 (1.14, 1.92)		1.62 (1.13, 2.32)	0.007
Educational qualification				
Less than primary	1		1	
Primary	0.88 (0.61, 1.27)	0.0062	0.89 (0.61, 1.29)	0.54
Middle/secondary	0.83 (0.53, 1.30)		0.68 (0.48, 0.96)	0.03
High secondary	0.52 (0.36, 0.75)		0.58 (0.38, 0.88)	0.01
Graduate and above	0.74 (0.38, 1.41)		0.83 (0.43, 1.58)	0.57
Marital status				
Unmarried	1	0.0864	-	
Married/live-in	1.27 (0.57, 2.81)			
Separated/divorced/widow/deserted	1.87 (0.80, 4.36)			
Wealth index				
Poorest	1	0.0588	-	
Poorer	1.03 (0.77, 1.37)			
Middle	0.83 (0.595, 1.15)			
Richer	1.03 (0.70, 1.52)			
Richest	1.51 (1.01, 2.26)			
Consumption of alcohol				
Does not consume alcohol	1	0.1337	1	
Occasionally	0.99 (0.77, 1.28)		1.34 (1.01, 1.80)	0.04
Weekly	0.67 (0.45, 0.99)		0.85 (0.52, 1.39)	0.53
Tobacco usage				
Do not consume tobacco	1		1	
Smoke tobacco	1.13 (0.87, 1.49)	0.1259	1.41 (1.08, 2.06)	0.02
Smokeless tobacco	0.90 (0.71, 1.16)		1.23 (0.93, 1.62)	0.15
Both smoke and smokeless	0.62 (0.35, 1.09)		0.69 (0.34, 1.43)	0.32
Health insurance				
No	1	0.9084	-	
Yes	1.02 (0.73, 1.41)			
Caste				
Scheduled caste	1	0.0629	-	
Scheduled tribe	0.71 (0.46, 1.07)			
Other backward class	1.14 (0.81, 1.59)			
Others	0.85 (0 .65, 1.11)			
Residence				
Rural	1	0.9183	-	
Urban	1.01 (0.79, 1.30)			
Duration DM				
<8 years	1		1	
≥8 years	1.34 (1.09, 1.78)	0.006	1.41 (1.07, 1.85)	0.015
DM medication				
No	1	0.6765	-	
Yes	1.06 (0.81, 1.39)			
Taking insulin shots				
No	Ref		1	
Yes	2.17 (1.28, 3.67)	0.0039	1.07 (0.67, 1.68)	0.77
Special diet for DM				
No	1	0.9868	-	
Yes	0.99 (0.793, 1.25)			

## Discussion

The present analysis from the large nationally representative LASI survey from India observed that oral morbidities were present in a majority of patients with DM, especially painful teeth, bleeding gums, and partial edentulism, suggestive of reduced quality of life. Compared to the general population, the prevalence of oral morbidities in patients with DM within the LASI dataset is substantially higher corroborating prior evidence of worsening oral health from underlying inflammation and infective conditions [21,22]. Furthermore, in this study, a greater duration of DM was associated with poor oral health, indicative of the deleterious effect of long-term DM on oral health status. Furthermore, the present study observed that the use of tobacco and alcohol was also associated with higher risk of oral morbidities, which is consistent with previous evidence, suggestive of them reducing salivary flow and increasing risk of damage to oral tissues [23-26].

In this study, patients with DM having less than graduate educational qualifications were significantly protected against oral morbidities compared to those with a higher educational status, a finding in contradiction to similar analysis in multimorbid individuals [27]. Consequently, healthcare providers should engage in oral health promotion in patients with DM towards improving both awareness and practices conducive to good oral hygiene.

Interestingly, results from this study indicate that patients with DM of female compared to male gender had significantly higher odds of having oral morbidities. However, previous studies show mixed direction association, with a study from Korea highlighting that the prevalence of males was higher as compared to females [28]. Nevertheless, biological plausibility of higher female susceptibility to oral health problems due to postmenopausal hormonal fluctuations exists, risk that can be further accentuated by comparatively poor oral health-seeking behavior of female patients in Indian health settings [29,30].

In this study, less than 1% of the respondents had visited a dentist in the past 12 months, while almost three in four patients with DM reported non-utilization of any OPD services in the past 12 months. A previous study in a tertiary care facility in Delhi, India, observed that patients with DM were unlikely to seek dental visits when asymptomatic or due to fatalistic health beliefs [19]. The poor oral health-seeking behavior observed in this study underscores the need for sustained oral health awareness campaigns and improved accessibility and affordability of oral health services. Integrating dental care into DM management programs and strengthening oral healthcare infrastructure are crucial steps to enhance overall health outcomes for patients with DM having oral co-morbidities. Policymakers must prioritize targeted preventive interventions to bridge the gaps in dental care utilization and address the burden of oral morbidities in DM patients. Collaboration between physicians, diabetologists, and dental practitioners is warranted for evolving patient-centered and comprehensive treatment strategies to improve oral health status and preservation of oral health.

The strengths of the study are the large sample size and a nationally representative sample, which improved the accuracy and representativeness of the study findings. However, there exist certain study limitations. First, this is a cross-sectional analysis, which prevents causality assessment, and panel data from future rounds of the LASI follow-up survey will enable ascertainment of the incidence and risk factors of oral morbidities in patients with DM. Second, the information about oral comorbidities and the use of medical services was provided voluntarily by the subjects through self-reporting, which is subject to recall and social desirability biases that reduce the validity of the study findings. Third, information on key variables, such as existing oral self-care practices, and specific barriers to oral health-seeking in vulnerable populations were lacking in this secondary database study, and thus future rounds of the LASI may incorporate additional variables relating to this major public health challenge.

## Conclusions

A majority of patients with DM in India have significant oral morbidities, while nearly two in three DM patients have partial edentulism, while most report negligible oral health-seeking behavior from dental healthcare professionals. Increasing duration of DM, concomitant use of tobacco/alcohol, female sex, and higher educational status are significant predictors of oral health morbidities in patients with DM. Future research should assess the feasibility of integration of oral healthcare services with existing diabetes care within outpatient settings to enhance patient qualify of health and improve health outcomes.
